# Identification of 27 allele-specific regulatory variants in Parkinson’s disease using a massively parallel reporter assay

**DOI:** 10.1038/s41531-024-00659-5

**Published:** 2024-02-27

**Authors:** Sophie L. Farrow, Sreemol Gokuladhas, William Schierding, Michael Pudjihartono, Jo K. Perry, Antony A. Cooper, Justin M. O’Sullivan

**Affiliations:** 1 Liggins Institute, The University of Auckland, Auckland, New Zealand; 2 The Maurice Wilkins Centre, The University of Auckland, Auckland, New Zealand; 3 Department of Ophthalmology, The University of Auckland, Auckland, New Zealand; 4 Australian Parkinsons Mission, Garvan Institute of Medical Research, Sydney, NSW Australia; 5 St Vincent’s Clinical School, University of New South Wales, Sydney, NSW Australia; 6 Singapore Institute for Clinical Sciences, Agency for Science Technology and Research, Singapore, Singapore; 7 MRC Lifecourse Epidemiology Unit, University of Southampton, Southampton, United Kingdom

**Keywords:** Epigenetics, Epigenetics, High-throughput screening, Genomics

## Abstract

Genome wide association studies (GWAS) have identified a number of genomic loci that are associated with Parkinson’s disease (PD) risk. However, the majority of these variants lie in non-coding regions, and thus the mechanisms by which they influence disease development, and/or potential subtypes, remain largely elusive. To address this, we used a massively parallel reporter assay (MPRA) to screen the regulatory function of 5254 variants that have a known or putative connection to PD. We identified 138 loci with enhancer activity, of which 27 exhibited allele-specific regulatory activity in HEK293 cells. The identified regulatory variant(s) typically did not match the original tag variant within the PD associated locus, supporting the need for deeper exploration of these loci. The existence of allele specific transcriptional impacts within HEK293 cells, confirms that at least a subset of the PD associated regions mark functional gene regulatory elements. Future functional studies that confirm the putative targets of the empirically verified regulatory variants will be crucial for gaining a greater understanding of how gene regulatory network(s) modulate PD risk.

## Introduction

The majority of people diagnosed with Parkinson’s disease (PD) are considered to have sporadic disease, caused by multiple different factors, each contributing a degree of risk. Genome-wide association studies (GWAS) are used to identify genetic risk variants, specifically single nucleotide polymorphisms (SNPs), that are strongly associated (*p* ≤ 5 × 10^−8^) with a disease or phenotype of interest. There have been three GWAS meta-analyses conducted in the last decade for PD^1–3^, the most recent of which compared 37,688 PD cases, 18,618 proxy cases, and 1.4 million controls. This study identified 90 risk variants across 78 genomic loci^3^. However, assigning target genes and functionality to these risk loci is problematic, given that 80 (89%) of the 90 PD-associated SNPs are located within non-coding genomic regions (intronic or intergenic). Linkage disequilibrium (LD) further complicates the situation as the tag GWAS SNP (i.e., the SNP with the smallest *p-*value within an identified disease-associated risk locus – not necessarily the causal SNP) is often strongly correlated with nearby variants, making it difficult to identify the causal SNP^4^. Despite these challenges, it is known that disease associated SNPs are enriched within gene regulatory elements^5^, indicating that one possible function of these SNPs may be to regulate gene expression^6^

In addition to GWAS loci, which have predominantly been identified through analyses involving individuals with the sporadic form of PD, there are a number of mutations that have been reported to cause PD^7^. Such mutations have been identified in genes such as *PINK1* and *LRRK2* and are typically associated with the familial, early onset form of PD. Although often referred to as causal, there remains significant uncertainty associated with many of these proposed monogenic PD genes^7,8^. Notably, the penetrance of the causative mutations within these genes is highly variable, and thus the question of whether there are other mechanisms (i.e., gene regulatory mechanisms; epigenetic modifications) influencing the impact of these mutations arises^9^. For simplicity, we will refer to the genes as ‘*PARK’* genes, but recognise this term should be used with caution.

**Fig. 1:**
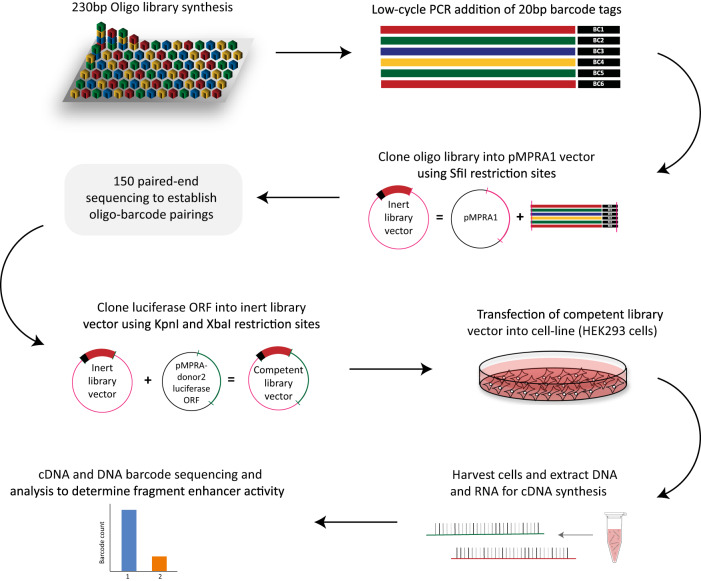
MPRA experimental workflow. Oligonucleotides (230 bp) were synthesised as a library and barcodes added (low-cycle PCR amplification). The library was subsequently cloned into pMPRA1 to create the inert library vector. The inert library was sequenced (150 paired-end sequencing; Illumina HiSeq X; 400 M read depth) to establish barcode-oligo pairings. The inert library was linearised between the barcode and oligo and a minimal promoter and luciferase open-reading frame (ORF) inserted by directional cloning to create the competent library. The competent library was transfected into HEK293 cells using Lipofectamine-3000. At 24-h post-transfection, DNA and RNA (for cDNA synthesis) were harvested using the Qiagen Allprep DNA/RNA extraction kit. DNA and cDNA were prepared for sequencing through PCR amplification and barcodes were then sequenced (Illumina HiSeq X). Allele-specific enhancer activity was determined using the *mpralm* R package^22^.

When considering the function(s) of both risk and causal variants, many computational prediction tools exist^10,11^. However, these tools typically have limited predictive utility, especially when used in isolation^12–14^. Alternatively, massively parallel reporter assays (MPRAs) are a high-throughput, in vitro tool that enable one to simultaneously test one putative function for thousands of variants - the existence of allele specific regulatory activity at each locus^15,16^. This assay takes advantage of the well-established luciferase reporter gene assay^17^, by which a synthesised library of barcode-tagged, putative regulatory sequences is cloned into a reporter plasmid, and transfected into the cell-line being used. High-throughput sequencing of the transcribed barcodes from the pooled cells can then be used to determine levels of enhancer activity for each locus (Fig. 1).

Here we used an MPRA to screen 5254 variants with known or putative connections to PD, to assess which of the variants exist within regulatory elements, and whether the PD risk variant allele modulates expression. We identified 138 putative enhancer elements in HEK293 cells, 27 of which were confirmed as allele-specific. Notably, 23 of the 27 allele-specific enhancers are predicted to disrupt transcription factor binding sites. For the vast majority of studied PD GWAS loci, the identified allele-specific enhancer element was not the original GWAS tag SNP. Furthermore, integrating expression quantitative trait loci (eQTL) and Hi-C data (across peripheral and CNS tissues) identified an average of 11 putative target genes per regulatory element identified by MPRA. Collectively, the results of this study provide insights into the regulatory potential of variants that are associated with PD risk. By assigning enhancer functionality to PD-associated variants, we provide a fine-mapped subset of variants that can be exploited further to gain a greater understanding of how the gene regulatory network potentiates risk in PD. In a complex, polygenic, disease such as PD, integrating data on the PD gene regulatory network with other ‘omic data types will be critical for generating personalised molecular profiles and developing robust patient stratification tools.

## Results

### Construction of PD-associated variant MPRA library

**Fig. 2:**
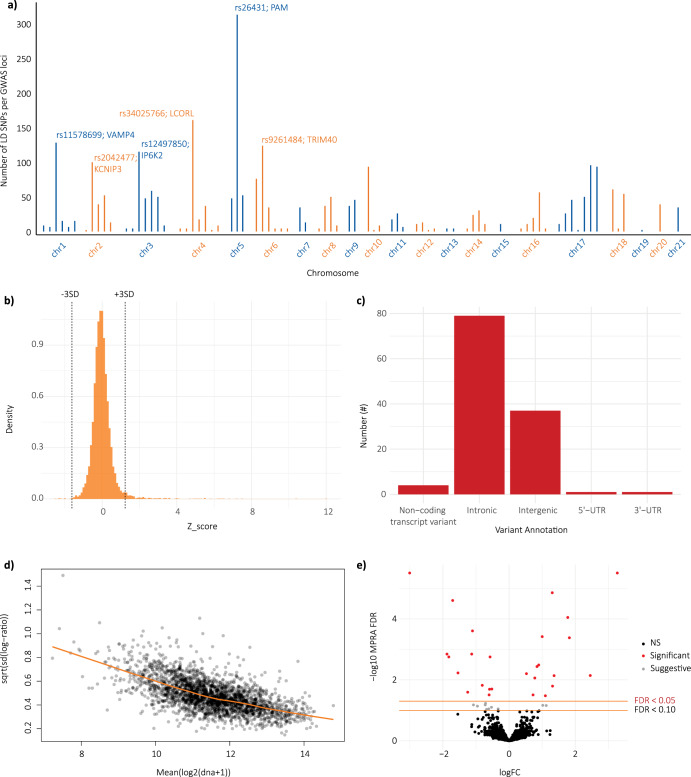
MPRA identifies 123 PD-related regulatory elements, 27 of which act in an allele-specific manner in HEK293 cell line. **a** Histogram showing number of LD SNPs tested for each of the 78 loci associated with PD by Nalls et al.; **b** Histogram showing the range of Z-scores of the putative regulatory elements. Dashed lines = mean +/−3 SD; **c** Variant annotation for the target SNPs within the 123 regulatory elements that were identified as enhancers (includes general and allele-specific enhancers; annotations from HaploReg v4.1); **d** Activity measures of putative regulatory elements, as calculated by *mpralm*. Activity is presented as the log2 ratio of aggregated RNA counts over aggregated DNA counts for all tested enhancers; **e** Volcano plot showing allelic regulatory activity of 4910 putative regulatory elements included within the library. Red dots indicate significant (FDR < 0.05) allele-specific enhancers, and grey dots indicate suggestive (FDR < 0.10) allele-specific enhancers.

We constructed an oligonucleotide library containing 10,484 elements (5254 allele pairs) that have been putatively linked to PD (Supplementary Table 1). The library included variants in strong LD (R^2^ > 0.8) with variants associated with PD in the three most recent meta-GWAS^1,3,18^ (Supplementary Table 1 [PD-GWAS LD; SNP in strong (R^2^ > 0.8) LD with a PD GWAS tag SNP]). An LD cut off of R^2^ > 0.8 was used for nominating variants linked to the tag GWAS SNPs. This method minimizes a priori assumptions, although there are more refined fine-mapping methods. Given the significant overlap between the three meta-GWAS studies^1,3,18^, the data presented is based on the most recent, and largest, GWAS meta-analysis conducted by Nalls et al. (Supplementary Table 2)^3^. On average, each PD-associated locus was represented by 35 SNPs (range: 1–315 SNPs; Fig. 2a). Common SNPs within 21 known *PARK* genes^7^ [*PARK* intragenic; SNP within a known *PARK* gene – list of *PARK* genes obtained from Blauwendraat et al. 2020], and distal variants associated with the expression of these *PARK* genes [*PARK* eQTL; SNP putatively associated with regulation of one of the *PARK* genes] were also included (Supplementary Tables 3 and 4). This extends our previous investigations of the regulatory network associated with *GBA*^19^. We also included 73 SNPs that are functional (i.e. ATAC-seq and H3K27ac ChIP-seq) in microglial cells^20^ (Supplementary Table 5), to enable an estimation of regulatory overlap across different cell types. A library of oligonucleotides (230 bp) was synthesised to centre on each variant of interest. Adapter sequences, including unique 20 bp barcodes, were added to the oligonucleotide library using a two-stage, low-cycle PCR. On average, each element within the library was mapped to 449 barcodes (range: 1–9333, Supplementary Fig. 1; Fig. 1 part 4). The putative enhancer elements were then directionally cloned upstream of a minimal promoter (pMPRA1/pMPRAdonor2), thus driving the expression of the luciferase reporter gene and enabling transcript quantitation of the tagging barcodes by RNAseq. This method is modified from Uebbing et al. ^21^ and Tewhey et al. ^16^.

We transfected the prepared MPRA library into HEK293 cells, performing a total of three technical replicates. DNA and RNA (for cDNA synthesis) were harvested, DNA and cDNA were prepared for sequencing through PCR amplification and barcodes were then sequenced (Illumina HiSeq X). Allele-specific enhancer activity was determined using the *mpralm* R package^22^. We observed strong correlation between the three replicates when comparing the composition and frequency of barcodes per element (r = 0.69–0.96; Supplementary Fig. 2). We had high coverage of the MPRA library, capturing 8849 of the 10,496 elements (81%) tested, 8548 of which mapped to at least 5 unique barcodes (Supplementary Figs. 3 and 4).

### Identification of PD-associated MPRA regulatory elements

We first sought to identify active elements within the MPRA library, irrespective of whether the enhancer activity was allele-specific (i.e., general enhancers). Aggregated RNA (cDNA) barcode counts were compared against the corresponding aggregated DNA barcode counts, and z-scores were calculated. Elements were designated as ‘general enhancers’ if they had a z-score of 3 or greater ($\pm$3 SD from the mean). Using this approach, we identified 138 general enhancers in HEK293 cells (Fig. 2b; Supplementary Table 6).

Regulatory elements that have allele-specific enhancer activity were identified using the *mpralm* R package^22^. Using this approach, we identified 27 elements that exhibited allele-specific regulatory activity, at an FDR cut-off of 0.05 (Fig. 2d, e; Table 1; Supplementary Table 7). 21 of the 27 elements were also identified as general enhancers, indicating that both alleles meet the threshold to be classed as enhancers, but one allele has a significantly greater regulatory effect (Supplementary Table 7 [column K]). It is likely that more of the identified regulatory elements are allele-specific but, in some instances, either the reference or alternate allele was not represented by sufficient barcodes for inclusion in the downstream analysis (Supplementary Table 8). Focussing solely on the 78 loci identified in the most recent PD GWAS meta-analysis^3^, we identified at least one regulatory variant (general and/or allele-specific enhancer) for 41% (32 out of 78) of the PD associated loci (Supplementary Tables 2, 6 and 7). This includes 10 loci (of 78) where we identified at least one allele-specific enhancer. Intriguingly, for some loci we identified multiple regulatory elements, consistent with findings from Abell et al. that demonstrate genetic association signals can arise from several tightly linked causal variants^23,24^. For example, for the chr3p21.31 GWAS locus (tagged by rs12497850), we tested 117 variants and identified 6 of these to be regulatory elements, one of which was allele-specific (rs6770112; FDR corrected *p* = 0.025).

**Table 1:** *mpralm* Allele-specific enhancers (FDR [adj. *P*.value] < 0.05)

Tag/representative SNP for GWAS loci	GWAS /other mapped gene(s)	SNP nomination category^a^	rsID^b^	logFC^c^	t^d^	*p*.value^e^	adj.*p*.val^f^	B^g^
	*DNAJC6*	*PARK* intragenic	rs6689005	0.7237	4.3200	0.000319078	0.031076676	0.2096
	*LRRK2*	*PARK* intragenic	rs2723264	2.4527	5.1134	4.97E−05	0.007183866	0.9203
	*PINK1*	*PARK* intragenic	rs7532202	−0.5914	−4.5400	0.000189986	0.020278954	0.7070
rs10463554/ rs26431	*PAM*	PD-GWAS LD	rs34788	−1.8215	−5.8955	8.37E−06	0.001765235	3.6351
rs10463554/ rs26431	*PAM*	PD-GWAS LD	rs62362545	0.7767	4.9850	6.70E−05	0.008660957	1.8011
rs11950533	*TXNDC15, C5orf24*	PD-GWAS LD	rs113661575	1.3042	8.9366	1.69E−08	1.38E−05	9.7516
rs12497850	*IP6K2*	PD-GWAS LD	rs6770112	−1.2506	−4.4274	0.000247688	0.025336401	0.5192
rs12600861	*CHRNB1*	PD-GWAS LD	rs55749333	−1.1298	−6.1038	5.26E−06	0.001421964	4.0717
rs1867598	*ELOVL7*	PD-GWAS LD	rs4700390	−1.8774	−6.0606	5.79E−06	0.001421964	4.0490
rs2904880	*RABEP2, CD19*	PD-GWAS LD	rs11646653	−3.0047	−10.0393	2.39E−09	3.11E−06	11.5308
rs3104783	*CASC16*	PD-GWAS LD	rs3104788	−0.5753	−5.8817	8.63E−06	0.001765235	3.4501
rs3104783	*CASC16*	PD-GWAS LD	rs11860998	1.3594	5.0838	5.33E−05	0.007265473	1.9448
rs34025766	*LCORL*	PD-GWAS LD	rs112525610	0.8431	5.4918	2.08E−05	0.003653424	2.8718
rs34025766	*LCORL*	PD-GWAS LD	rs6449345	1.3133	4.6793	0.000136951	0.016010267	1.1253
rs4954162/ rs57891859	*TMEM163*	PD-GWAS LD	rs3739034	−1.7047	−8.4702	4.04E−08	2.48E−05	8.7887
rs4954162/ rs57891859	*TMEM163*	PD-GWAS LD	rs16830920	1.8216	6.7294	1.35E−06	0.000414713	5.4550
rs9261484	*TRIM40*	PD-GWAS LD	rs3815082	1.0003	6.8322	1.09E−06	0.000380922	5.6154
rs9261484	*TRIM40*	PD-GWAS LD	rs1076229	0.5242	5.1996	4.08E−05	0.006252808	2.1011
	*GIGYF2*	*PARK* eQTL	rs812383	3.2699	10.0055	2.53E−09	3.11E−06	11.1122
	*TMEM230*	*PARK* eQTL	rs8121449	1.7729	7.6989	1.82E−07	8.93E−05	7.4150
	*C2orf82*	*PARK* eQTL	rs6719061	−1.1065	−7.1117	6.04E−07	0.000247042	6.2727
	*VPS13C*	*PARK* eQTL	rs78222414	0.8950	5.5766	1.72E−05	0.00324324	3.0401
	*ATP13A2*	*PARK* eQTL	rs2746478	−1.5447	−5.2525	3.61E−05	0.005903798	2.2690
	*POLG*	*PARK* eQTL	rs7161856	−0.8109	−4.7258	0.000122782	0.015071546	1.1188
	*DNAJC6*	*PARK* eQTL	rs208376	−0.5213	−4.5660	0.000178715	0.019943008	0.7345
	*TMEM230*	*PARK* eQTL	rs6084993	−0.6038	−4.3069	0.000329122	0.031076676	0.2192
	*VPS13C*	*PARK* eQTL	rs11071650	1.0959	4.2631	0.000364996	0.033187599	0.2536

^a^SNP nomination category indicates how the regulatory element was initially linked to PD: ‘*PARK* intragenic’ = SNP within a known *PARK* gene; ‘PD-GWAS LD’ = SNP in strong (R^2^ > 0.8) LD with a PD GWAS tag SNP (tag SNP highlighted in first column); ‘*PARK* eQTL’ = SNP putatively associated with regulation of one of the *PARK* genes (specific gene indicated in second column).

^b^SNP in which the 230 bp putatively regulatory element was centred on.

^c^log fold-change (changes in activity) between the reference and alternate allele.

^d^t-statistic for RNA count difference between reference and alternate allele.

^e^*p*-value for calculated t-statistic.

^f^FDR correct *p*-value, only elements with FDR *p*-value < 0.05 are reported.

^g^B-statistic, the log-odds of differential expression.

### Functional annotation of MPRA identified regulatory elements

**Fig. 3:**
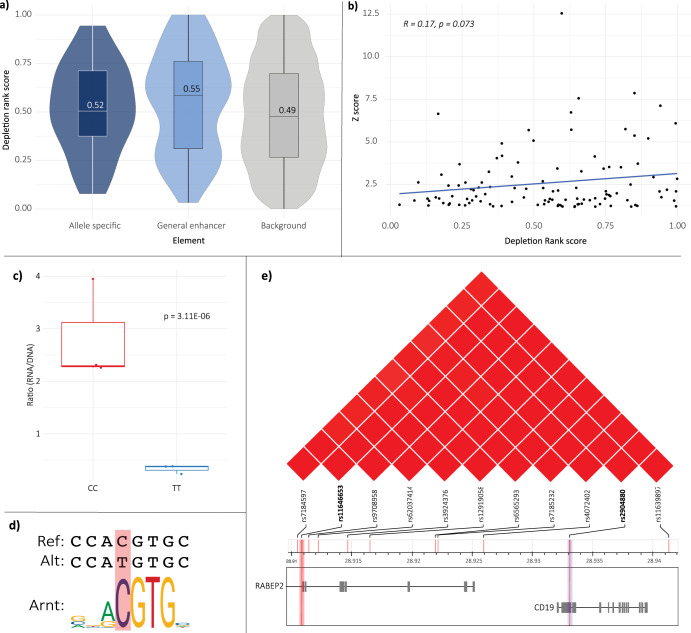
Characterisation of MPRA-identified regulatory elements. **a** DR score for variants included in the MPRA library. DR scores are plotted according to whether the variant marks an allele-specific enhancer, general enhancer, or non-enhancer/background variants within the MPRA library. MPRA-identified regulatory elements have higher depletion rank scores when compared to non-enhancer elements within the library. **b** There was a direct correlation between Z-score and DR score for identified enhancer variants. **c** Presence of the reference allele at rs11646653 is associated with significant enhancer activity (*p* = 3.11E−06). **d** Presence of the alternate allele at rs11646653 disrupts the Arnt transcription factor binding site, consistent with weakened enhancer activity. **e** rs11646653 is identified as a strong allele-specific enhancer within the PD-GWAS chr16 loci, originally tagged by the rs2904880 GWAS SNP. Figure adapted from LDLINK browser^81^.

The MPRA regulatory elements we identified were largely within intronic and intergenic regions (Fig. 2c). Regions of functional importance can be identified using depletion ranks (DR) as a measure of sequence conservation for 500 bp genomic windows. Regions are ranked with a score from 0 to 1 (0 being most depleted, i.e., most constrained)^25^. Halldorsson et al. previously demonstrated that non-coding regions (and regions containing GWAS variants) represented the majority of regions under sequence constraint, and thus have low DR scores^25^. The mean DR score for all variants included in the MPRA library was 0.49. The enhancer variants we identified had, on average, higher DR scores (general [0.55] and allele-specific [0.52]) when compared to all other variants included in the MPRA library (Fig. 3a). Although this finding was somewhat unexpected, this may in part be due to the fact that the majority of the enhancer variants are intergenic and would be considered distal enhancer-like sequences. Finally, lower z-scores (i.e., weaker enhancers, calculated from Fig. 2b, Supplementary Table 9) weakly correlated with lower DR scores (indicative of variant depletion), consistent with selection against nucleotide variation at these enhancers (Fig. 3b).

FABIAN^26^ was used to identify if the variants are predicted to disrupt transcription factor binding sites (TFBS). We limited our analysis to use only transcription factor flexible models (TFFMs) for TFBS disruption prediction, as they have been shown to outperform position weight matrices (PWMs)^26,27^. FABIAN provides one score per TFBS per variant, from 1 to −1, with 0 indicative of no disruption. We chose an arbitrary cut-off of $\pm$0.8 to select ‘high confidence’ predictions. Using this threshold, we found that 23 out of 27 MPRA allele-specific enhancers (FDR < 0.05) are predicted to disrupt at least one TFBS (Table 2; Supplementary Table 10). Several of the allele-specific enhancers are predicted to disrupt (negative score) or create (positive score) multiple TFBS (i.e., rs6689005, rs2723264 etc.), indicating that these SNPs may have significant functional implications in terms of gene regulation, consistent with the notion that these were identified to be significant enhancers. Finally, given the relatively small number of identified allele-specific enhancers, we did not identify enrichment for any specific TFs whose binding motifs are disrupted by MPRA regulatory variants.

**Table 2:** 23 of 27 allele-specific enhancers (FDR < 0.05) disrupt or create at least one transcription factor binding site

rsID	Chr. Position (GRCh38)	Transcription factor(s)	Allele-specific enhancer FDR
rs2746478	chr1:17015765	GLIS3,ZIC5	<0.05
rs6689005	chr1:65375651	SREBF1,EHF,ELF1,ELF4,ERF,ERG,ETS1,ETS2,ETV1,ETV2,FLI1,GABPA,IKZF1,STAT2	<0.05
rs16830920	chr2:134707896	HSF2	<0.05
rs3739034	chr2:134725811	EOMES,GATA1,GATA2,GATA4,GATA6	<0.05
rs6719061	chr2:232831175	PRDM14,TRPS1	<0.05
rs812383	chr2:232872422	NKX3-2,TBX19	<0.05
rs78222414	chr2:73977449	ZBTB26,ZBTB6	<0.05
rs6770112	chr3:49136573	ZNF135	<0.05
rs6449345	chr4:17932771	FOXH1	<0.05
rs112525610	chr4:17953199	EOMES,ZIC2	<0.05
rs62362545	chr5:103011748	HOXC10,POU2F1	<0.05
rs113661575	chr5:134594467	ZNF135	<0.05
rs4700390	chr5:60786718	E2F1,E2F4	<0.05
rs3815082	chr6:30146178	STAT5A,STAT5B,ZNF189	<0.05
rs2723264	chr12:40258718	BHLHA15,ISL1,NEUROD1,NEUROG2,MAFB	<0.05
rs11071650	chr15:62052408	ZBTB6,ATF1,ESR2,FOS,FOSL1,FOSL2,JUN,NFE2,NFE2L2,RORC	<0.05
rs7161856	chr15:89310952	SIX2	<0.05
rs11646653	chr16:28910828	ARNT,BHLHE40,BHLHE41,ZNF75D	<0.05
rs11860998	chr16:52594506	PRDM14	<0.05
rs55749333	chr17:7468613	RXRA	<0.05
rs8121449	chr20:5057712	ZNF416	<0.05
rs6084993	chr20:5084447	SIX2,TEAD1,TEAD2,TEAD3,TEAD4,NR1D1,PPARG,ZNF135	<0.05
rs208376	chr20:54006278	POU2F1,POU2F2,POU2F3,POU3F1	<0.05
rs12755229	chr1:65348550	NFATC1,NFATC2,STAT3,ZBTB26	<0.1
rs10929159	chr2:236024319	ESR1,ESR2	<0.1
rs34378	chr5:103064805	ZFP57	<0.1
rs10471496	chr5:60772520	ZNF692	<0.1

Full data, including directionality, can be found in Supplementary Table 10. The allele-specific enhancer FDR scores are taken from Supplementary Table 7, and are originally derived from the mpralm analysis.

We utilised HaploReg^28^ to identify overlaps between all identified enhancer elements and epigenetic marks, and to further characterise the identified enhancers (Table 3; Supplementary Table 11). All of the identified enhancer SNPs lie in intronic or intergenic regions, except for one SNP (rs11555596), which lies in the 3’UTR of the *VPS13C* gene. Some of the identified intronic enhancers may rather be acting as putative, cell-type specific, alternative promoters^29^. When combining allele-specific and general enhancers and comparing to all other elements within the MPRA library, there was no significant difference observed in their overlap with promoter or enhancer histone marks. We did, however, observe significant enrichment for overlap with DNase I hypersensitivity regions (*p* = 0.046) and protein binding sites (*p* < 0.01; ENCODE ChIP-seq data) in the enhancer group. Consistent with previous knowledge, these findings indicate that enhancer elements are more likely to be found in regions of open chromatin, and a subset of the enhancer SNPs may be driving regulatory effects by disrupting the binding of specific proteins. Despite this, the lack of enrichment for promoter or enhancer marks highlights the notion that epigenetic annotations alone cannot be used to predict enhancer elements from non-regulatory elements^30^.

**Table 3:** Overlap between MPRA elements and epigenetic marks

	Promoter element^a^	% of total	Enhancer element^b^	% of total	DNase I hypersensitivity^c^	% of total	Protein binding^d^	% of total
Allele-specific enhancers	1	3.70	16	59.26	9	33.33	2	7.41
General enhancers	22	24.18	46	50.55	37	40.66	23	25.28
Background MPRA elements (i.e., non-enhancer elements)	856	16.80	2765	54.28	1529	30.02	616	12.09
Proportion test *p* value allele vs general vs background^e^	0.033		0.678		0.085		<0.01	
Proportion test *p* value all enhancers vs. background^f^	0.518		0.779		0.046		<0.01	

^a^Number of elements overlapping with ChromHMM^82^ states corresponding to promoter elements.

^b^Number of elements overlapping with ChromHMM states corresponding to enhancer elements.

^c^Number of elements overlapping with DNase I hypersensitivity data peaks (narrowPeak algorithm).

^d^Number of elements overlapping with protein binding sites, data obtained from ENCODE Project ChIP-Seq.

^e^Proportion test comparing between the three separate sub-groups (i.e., allele-specific vs. general enhancer vs. background).

^f^Proportion test comparing between all enhancers (i.e., allele-specific + general enhancer) vs. background. Background = all elements in the MPRA library not identified to be regulatory. Data were obtained from HaploReg V4.

We next sought to investigate the likely regulatory activity of identified allele-specific enhancers within brain tissues and cell-types. Epigenetic annotations (i.e., open chromatin; histone modifications) are known to be indicative of enhancer or promoter activity. By analysing these annotations across a number of different datasets (FOUNDIN-PD ATACseq and methylation data (iPSC-derived dopaminergic neurons)^31^; epigenetic characterisation by Nott et al. ^32^*;* Fullard et al. ^33^; and Corces et al. ^34^) we aimed to determine whether the MPRA identified allele-specific enhancers (in HEK293 cells) are also active within brain cell-types or regions. 16 of the 27 (59%) identified allele-specific enhancers showed overlap with at least one epigenetic annotation from the aforementioned datasets (Supplementary Fig. 5). In addition, several showed significant overlap with multiple epigenetic annotations including: rs812383; rs4820323; rs2723264; rs113661575; rs1076229. Furthermore, through using GTEx^35^, we found that 19 of the 27 allele specific enhancer SNPs are acting as eQTLs in at least one of the thirteen brain tissues captured within the catalogue. Given our assay was completed in a HEK293 cell line, one would not expect all identified allele-specific enhancers to overlap with functional annotations in brain-related cell-lines. Nonetheless, the observed overlap highlights that many of the identified enhancers likely also act as enhancers within relevant brain regions, although further functional validation would be required to confirm such activity.

### GWAS tag SNP ≠ identified enhancer variant(s) within PD GWAS loci

We identified enhancer variants for 32 of the interrogated GWAS loci (which included 148 GWAS tag SNPs^1,3^; Supplementary Tables 2, 6 and 7). However, only three of the enhancer variants were also classified as a GWAS tag SNP (rs2740594, rs7938782, rs8005172). rs7938782 was identified as a general enhancer (non-allele-specific) and was the only PD GWAS associated SNP labelled as a tag SNP in Nalls et al. PD meta-GWAS^3^. The other two enhancer tag SNPs (i.e., rs2740594 and rs8005172) were identified as being associated with PD (as the tag SNPs) in an older PD meta-GWAS conducted by Chang et al. ^1^.

We sought to explore the profiles of MPRA identified regulatory variants that are in strong LD with the original GWAS tag SNPs. One such example falls within the *CD19* coding region which was marked by the tag SNP rs2904880 (Fig. 3c–e). In this instance, rs11646653 (LD: R^2^ = 0.867 with rs2904880) was identified as a strong allele-specific enhancer in the HEK293 cells (adj. *p* = 3.11E−06; Fig. 3c). This is consistent with ENCODE data which identifies rs11646653 as falling within a *cis*-regulatory element (combined from all cell types)^36^. Notably, the alternative allele T at site rs11646653 disrupts *Arnt* transcription factor binding (Fig. 3d; Table 2; Supplementary Table 10), hence weakening the enhancer activity. The DR score for rs11646653 was low (0.166), indicating a relatively high degree of constraint, and thus functional potential. Finally, integration of chromatin structure and eQTL data (across both peripheral and CNS cell lines/tissues) identified a number of potential target genes for rs11646653, including *NFATC2IP* and *SH2B1* (Supplementary Table 12, see methods). Further functional characterisation is required to pinpoint the exact effects of this locus in a PD-relevant cellular model.

### Previously identified microglial enhancer elements are not active in HEK293 cells

We included 73 loci (146 elements accounting for reference and alternate alleles) within the MPRA library that were previously identified as ‘Parkinson’s disease SNPs of interest’, due to their regulatory potential in microglia (Supplementary Table 5)^20^. Booms et al. took the 6749 SNPs of interest from the most recent PD meta-GWAS^3^ and overlapped the SNPs with regulatory epigenetic marks to identify functional PD SNPs in microglia^20^. Here, we determined whether this regulatory potential was microglia-specific or was also captured in a more generic, HEK293 cell line. None of the 73 loci were allele-specific enhancers in HEK293 cells, although 4 of the 73 loci were located within general enhancer regions (non-allele specific) based on Z-score (Supplementary Table 6). These 4 loci overlap with H3K4me3 and marks across multiple cell-types (ENCODE data; Supplementary Table 11), indicating that these regions are likely to be ubiquitous promoters or enhancers, as opposed to cell-type specific. Therefore, we conclude that the remaining 69 SNPs of interest^20^ are more likely to be cell-type specific enhancers in microglia. However, functional reporter assays within a microglial cell line should be conducted to confirm this.

### Precise editing of identified allele-specific enhancer variants confirms gene expression regulatory role(s) in the endogenous environment

For three of the identified allele-specific enhancer variants (rs11646653, rs78222414; rs3815082) we used CRISPR-cas9 technology to create isogenic pairs of cell-lines (induced pluripotent stem cell [iSPC] – KOLF2.1J cell line^37^) that differ at the base of interest. We confirmed the absence of off-target effects using in silico prediction tools^38^ and Sanger sequencing in cases where the guide RNA had 3 or less mismatches. We then completed RNAseq to identify differentially expressed genes associated with the single base change in the undifferentiated iPSC state. All three of the explored variants led to a large number of differentially expressed genes when comparing WT replicates to the edited clones. Many of the differentially expressed genes are consistent across all three sets of edits, suggesting that the expression of these genes may be altered due to the nucleofection process. We have therefore focused on the differentially expressed genes that are unique to each target (Supplementary Table 13; Supplementary Fig. 6). On average, each introduced SNP was associated with 260 differentially expressed genes (log2FC > 1.0) across three replicates. Of note, this analysis showed that the alternate genotype compared to wild-type at rs78222414 led to differential expression of *VPS13C*, as predicted from previous analyses (described below in the ‘Putative distal regulators of PARK genes’ section; Supplementary Table 12). Through completing this work, we demonstrated that all three variants are indeed acting as enhancers in the native chromatin context and affect the expression of a number of genes.

### Assigning target genes to MPRA regulatory elements

**Fig. 4:**
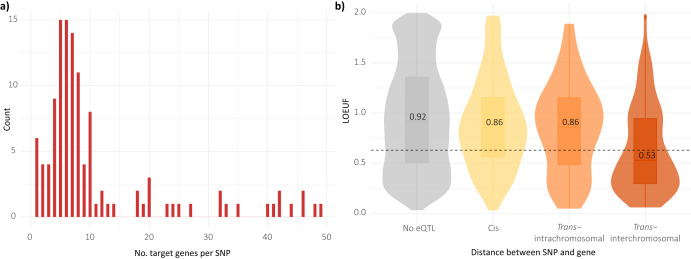
Overlap of MPRA identified regulatory SNPs with eQTLs and target genes. **a** Number of putative target genes per MPRA identified enhancer element (SNP), identified through use of CoDeS3D algorithm; **b** Genes that are loss of function intolerant, as measured by a continuous LOEUF score, are enriched in *trans*-regulatory interactions. Median LOEUF scores for each category are presented on each violin plot. The LOEUF score is a continuous value that indicates the tolerance of a given gene to inactivation. Low LOEUF scores indicate stronger selection against loss-of-function variation. Dotted line indicates the mean LOEUF score (0.63) for 678 genes that are deemed essential for human cell viability.

Combining data on chromatin architecture with gene expression data has proven to be an effective approach for the translation of variant to target gene^39–41^ (i.e., the gene(s) being regulated by the variant). We have previously integrated spatial (Hi-C) data with gene expression (expression quantitative trait loci; eQTL) data, to identify putative target genes of PD GWAS tag SNPs^40^. We used CoDeS3D^39^ to link the MPRA regulatory elements (includes general enhancers and allele-specific enhancers) to putative target genes across all tissues (Supplementary Table 12). We identified an average of 11 target genes (mean; range 1 – 49 target genes) for 133 of the 138 regulatory elements (Fig. 4a). Only 5 SNPs (rs80126945, rs11928552, rs16830920, rs572955542, rs11175655) had no identifiable target genes using this approach. In comparison to previous analyses exploring GWAS loci^40,42^, MPRA identified regulatory SNPs were significantly enriched for eQTLs (proportion test, *p* < 0.01), consistent with the recognition that tag SNPs in GWAS are frequently not the functional elements. The two SNPs (rs1076229 and rs9261504) with the most target genes were both SNPs in linkage with the same PD-GWAS tag SNP (rs9261484) and are located within the HLA locus (Chromosome 6p21.3). Other target genes of note include: ARHGAP27, GPNMB, KANSL1, KAT8, PRSS38 and STX4, all of which were deemed to be causal for PD by Mendelian Randomisation (MR) analysis^43,44^. Our analysis identified regulatory variants that are putatively associated with the expression of these genes that are causal for PD, and thus there is strong rationale for future functional studies to understand these regulatory interactions further.

Finally, we tested whether the putative target genes of the MPRA identified regulatory SNPs were more likely to be intolerant to loss-of-function variation than the background set of all genes (i.e., all genes listed in gnomAD). This links in with the notion that the expression of highly constrained genes is more likely to be altered through subtle regulatory changes, when compared to genes that are not highly constrained^45,46^. The LOEUF score (range: 0–2) is used to determine the level of constraint, with 0 indicating depletion of loss-of-function variation (i.e., highly constrained; essential gene), and 2 indicating minimal constraint^46^. In comparison to background genes, the target genes were significantly more intolerant to loss-of-function variation (t-test; *p* < 0.01). This intolerance was predominantly driven by genes regulated through trans-interchromosomal interactions (Fig. 4b), consistent with previous observations made by our group^40,42,47^.

### Putative distal regulators of PARK genes

**Fig. 5:**
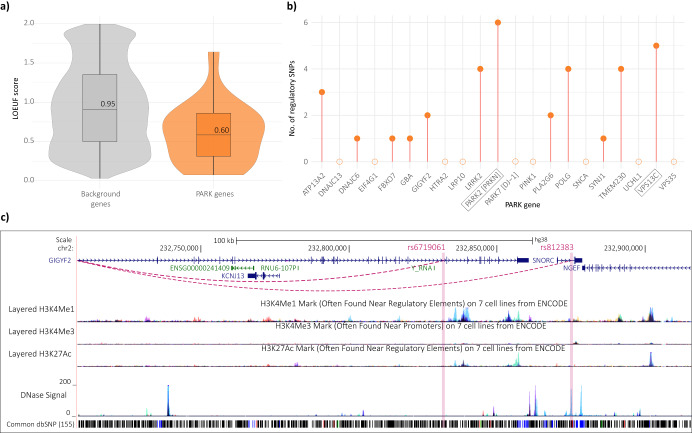
*PARK* genes are depleted for loss-of-function variation, and thus expression of these genes may be regulated through distal interactions. **a** LOEUF score of the 21 *PARK* genes vs. LOEUF score of background genes; **b** Number of identified enhancer SNPs regulating each PARK gene; **c** Two regulatory SNPs (rs6719061 and rs812383) are putatively associated with the expression of *GIGYF2* through *cis-* interactions, adapted from UCSC browser file.

The ‘*PARK’* genes included within this analysis (Supplementary Table 3) were initially outlined by Blauwendraat et al. as a list of monogenic PD genes. Although several of these genes are often denoted as *PARK* genes, for many there remains significant uncertainty around their reported causal associations with PD. As stated earlier, the *PARK* annotation was used for simplicity in this study. *PARK* genes have a mean LOEUF (loss-of-function observed/expected upper bound fraction) score of 0.612 (range = 0.074–1.641; Fig. 5a; Supplementary Table 3)^46^, indicating that these genes tend to be mutationally constrained. This is consistent with reports that mutations within these genes are deemed ‘causal’ for PD^7^. However, despite being labelled as causal, many of the mutations display incomplete penetrance, indicating that not everyone with the mutation will develop PD^48^. We hypothesised that there are other variants that modify the causative disease mutations by altering the expression of these disease-associated genes. We identified potential regulatory variants using CoDeS3D (as previously described^19^), and tested the enhancer activity of these variants within the MPRA. Of note, none of these variants have previously been associated with PD by GWAS. We identified putative enhancer variants (range 1–6) for 12 of the 21 *PARK* genes within HEK293 cells (Fig. 5b). For the remaining 9 *PARK* genes, we identified no variants with enhancer activity.

*GIGYF2* (Chr2q37.1) is depleted for loss-of-function variation (LOEUF = 0.077) and specific mutations within this gene are reported as causative of PD^49,50^. In our analysis, we identified two allele-specific enhancers that are associated with the expression of *GIGYF2*, one of which is intronic to *GIGYF2* (rs6719061), and the other (rs812383) lies upstream within intron 1 of *SNORC* (Fig. 5c). rs812383 lies within a DNase hypersensitivity region and overlaps a number of histone marks indicative of enhancer activity (Supplementary Table 11). The rs812383-*GIGYF2* eQTL regulatory interaction in the brain cortex is reported in both the GTEx^35^ and MetaBrain^51^ databases. Given the regulatory potential of these variants, it is possible that they mark alternative promoters, as opposed to being intronic or intergenic enhancers. However, discriminating these possibilities requires further investigation.

A group of four variants in high LD (R^2^ = 1) within intron 5 of *TEX2* (chr17) are associated with the expression of *PRKN* (*PARK2;* Supplementary Fig. 7) on chromosome 6. Unfortunately, for three of the four identified enhancer variants (rs9889475, rs9915286, rs9915598), either the ref- or alt- allele element was not represented by a satisfactory number of barcodes within our MPRA library. Therefore, we cannot infer whether the enhancer activity is allele-specific at these sites. Both the ref- and alt- allele elements were represented for the fourth variant (rs2166291), but no significant allelic difference was observed, only general enhancer activity.

## Discussion

Assigning function to disease-associated variants is a major challenge currently faced in the field of translational genomics, with the vast majority of GWAS-identified variants located within non-coding regions of the genome^52^. Although challenging, it is critical to understand where the disease risk is originating from and how these disease-associated variants potentiate risk, in order to advance our understanding of disease mechanism(s) and identify potential therapeutic targets. MPRAs were developed as a tool to assess the regulatory function of such variants and distinguish (causal) regulatory variants from those in strong linkage, potentially resolving a limitation that is inherent in genetic association studies. Here, we employed an MPRA to systematically evaluate the regulatory potential of 5254 PD-associated variants, identifying 138 general enhancers, including 27 allele-specific enhancers within HEK293 cells. 23 of the 27 allele-specific enhancers disrupt at least one TFBS, with many disrupting multiple TFBS. In addition to disruption of TF binding, there are likely other mechanisms through which the elements may be regulating gene expression, including: overlap with signature epigenetic markers (i.e., histone modification) or alterations in chromatin accessibility^52^.

The bulk of the elements included in our MPRA library were variants in strong LD within PD GWAS loci. For the majority of these loci, the GWAS tag SNP was not identified to be located within a regulatory element. This is consistent with previous studies exploring the regulatory potential of tag SNPs vs. those in LD^11^, and highlights the need for functional assays (e.g. MPRA) prior to downstream analyses. Furthermore, for several loci we identified multiple regulatory variants within a locus, consistent with recent findings from Abell et al. ^23,24^. The presence of multiple regulatory variants within a single risk-locus opens the possibility that the risk is due to the combined effects of changes within two or more control elements. For example, the PD GWAS meta-analysis that identified the locus tagged by rs57891859^3^ also identified rs4954162 as a potential tag-SNP, but it did not pass the final quality control. Both rs57891859 and rs4954162, and 52 SNPs in LD with either one or both of these SNPs, were included in our MPRA. We found that neither of these tag SNPs acted as enhancers in HEK293 cells. However, two SNPs (rs3739034 and rs16830920) in linkage act as allele-specific enhancers. rs16830920 is rare (MAF < 0.01) and thus we could not check for eQTL targets for this SNP. However, we identified a number of putative target genes for rs3739034, including *CCNT2* and *TMEM163*. This not only adds to previous association studies that have highlighted these genes as likely targets of this locus^3,53,54^, but also highlights a potential enhancer SNP within the locus that may drive the observed association. Of note, the reference allele at rs3739034 is acting as the enhancer, with the presence of the alternate allele weakening enhancer activity. This is consistent with the finding that the alternate allele disrupts binding of a number of GATA transcription factors, thus providing a potential mechanism through which weakening of the enhancer activity likely occurs. Future functional studies (i.e., CRISPR substitution) will be important to determine the synergistic effects of rs3739034 and rs16830920, and to advance our understanding of loci with multiple regulatory elements.

Beyond elucidating the regulatory activity of disease-associated variants, characterising the gene targets of these variants is key for understanding the overarching gene regulatory network, and for identifying potential therapeutic targets. We identified both proximal and distal putative gene targets for the regulatory variants (Fig. 4) using an approach that integrates Hi-C spatial data with gene expression eQTL data. A high proportion (133 of 138) of the MPRA enhancer variants were identified as spatial-eQTLs, indicating that the vast majority of these regulatory variants are impacting the expression of at least one gene.

Our approach also identified a small number of candidate regulatory variants that are putatively linked to the expression of causal *PARK* genes. Although known coding mutations occurring within this set of genes are deemed to be causal for PD, they typically display incomplete penetrance, suggesting there may be other modifying mechanisms. In addition, there is significant debate surrounding whether *GIGYF2* is actually a causal *PARK* gene, largely due to the fact that the monogenic links first identified in 2008^49^ have never been firmly corroborated^50,55^. Nonetheless, as aforementioned, we identified two allele-specific enhancers associated with altered *GIGYF2* gene expression, one of which is located distally (~11 kb upstream) to *GIGYF2*. We propose that these distal regulatory variants may either 1) modulate the expression of the target *PARK* gene to either amplify or dampen the effect of the causal mutation, or 2) interact with known mutations within these *PARK* genes. In summary, our analyses suggest that these identified regulatory variants are putatively associated with the expression of these genes, many of which act through *trans-*interactions. Moving forward, it will be important to consider whether the identified SNPs are associated with the penetrance of monogenic PD in mutation carriers. One method to explore this is through GWAS, to identify genetic variants that may modify the penetrance of causal PD mutations^56,57^. Although our method identified different variants to GWAS’s conducted for *GBA* and *LRRK2*^56,57^, it is probable that penetrance of these ‘causal’ mutations is dependent on an individual’s total background polygenic risk^58^, in combination with environmental factors. Compiling the results gained from these different studies (using novel methods) may be beneficial for informing future clinical trials to identify individuals most likely to respond to targeted, genetics-informed, treatments. Finally, in addition to population-level studies, functional studies may also be useful to confirm the associations identified in our study and to explore any epistatic interactions that may be occurring with reported causal mutations.

It is important to acknowledge the limitations associated with the MPRA based analysis we undertook. Firstly, this MPRA was conducted in HEK293 cells, due to the need for high transfection efficiency to enable adequate library coverage. This likely limits the generalisability of the assay for PD. Nonetheless, HEK293 cells are commonly used in PD research due to their robustness and amenability to transfection^59^, and, in addition, Yonatan Cooper and colleagues recently showed strong overlap of active regulatory regions between HEK293T cells and brain tissues^60^. One may also argue that using a more generic, or representative, cell type may be beneficial for the identification of more ubiquitous regulatory elements. Nonetheless, future studies would be warranted to compare the PD-associated regulatory landscape across different cell types and developmental stages. Secondly, our data processing and alignment methods were stringent, meaning a considerable amount of data was omitted and it is likely there are more allele-specific enhancers that were not identified because of this. Finally, there are several more generic limitations associated with the MPRA method itself. These include: sequence length of regulatory element within library; episomal vector environment as opposed to genome-integrated^21^; and weak regulatory effects that do not meet the required level for detection.

The sequence length of the regulatory elements represents not only a limitation of the MPRA method but also of the current SNP based approaches and utility of short-read sequencing methods. GWAS have been highly informative for identifying disease-associated risk loci and SNPs, but typically do not capture structural variants (SVs) or repeat expansions in the genome. Despite SNPs being the best characterised form of variation, it is likely that larger genomic events contribute to at least some of the identified GWAS signals and may indeed have greater impact on gene expression^61^. However, short-read sequencing and techniques such as MPRA cannot typically capture these forms of variation, and thus it has been hard to depict the contribution of such variants to disease risk^61^. The fast-growing application of long-read sequencing, and inference using short-read sequencing, is beginning to provide insight into the impacts of other forms of variation^62,63^. Moving forward, it will be important to consider the functional implications of identified SVs associated with PD, and also potential interaction and/or epistatic effects occurring between SVs and SNPs^64^.

The integration of our findings with further functional assays, such as CRISPR interference assays^60,65^, will strengthen our mechanistic understanding of the identified allele-specific enhancer variants within their native genomic context. In terms of PD, a disease where relatively little is understood about the genomic risk loci, these findings will be crucial for gaining a greater understanding of how the regulatory network potentiates risk in PD. Ultimately, gaining a mechanistic understanding will enable us to utilise the validated disease-associated variants in therapeutic target selection and for patient stratification, especially when considering genetically informed drug trials.

## Methods

### MPRA overview

The MPRA framework is very adaptable and has been used to study a multitude of different genetic elements, including enhancers^21,66,67^ & silencers^68^, splicing^69^, and protein translation^70^. The basic principle of the assay is that candidate regulatory elements are paired with unique barcodes and cloned into a reporter plasmid. Expression is measured by normalising reverse-transcribed RNA (cDNA) barcode counts against DNA barcode counts^71^. For this study, with the purpose of identifying allele-specific enhancer elements, the methodologies presented by Uebbing et al. ^21^. and Tewhey et al. ^16^. were used and adapted (Fig. 1).

### Variant selection & library design

To construct the oligonucleotide library, 5254 variants (SNPs) were selected (Supplementary Table 1). The oligonucleotide library included positive controls of ‘ubiquitous enhancers’ from the FANTOM dataset^72^ and random scrambled sequences as negative controls. The included SNPs are linked to PD either through GWAS^1,3,18^ (and linkage [R^2^ > 0.800]), or through association with the known *PARK* genes (e.g^19^; see ‘Identification of *PARK* gene eQTLs section below’). An additional set of 73 SNPs were included, due to their assignment by Booms et al. as functional SNPs in microglial cells, as determined by both ATAC-seq and H3K27ac ChIP-seq^20^. For every SNP, sequences were included containing both the reference and alternate alleles, with the variant of interest centred within the surrounding 200 bp of genomic sequence. For every 200 bp fragment, an additional 15 bp adapter sequence ([5’ adapter: ACTGGCCGCTTGACG]; [3’ adapter: CACTGCGGCTCCTGC]) was included on the 5’ and 3’ end, respectively. The final oligonucleotide library was synthesised by Agilent Technologies.

### Library backbone preparation

#### Inert library

The oligo library was amplified using a two-stage low-cycle PCR (MPRA_untailed primer pair followed by MPRA_SfiI_tailed primer pair), which enabled the incorporation of 20 bp long barcode tags (N_20_ where N = A, T, C, G with equal chance of incorporation) into the library, as well as the addition of required restriction sites. The amplified oligo library and pMPRA1 vector were then digested with *Sfi*I and ligated to form the inert library backbone. Following transformation and purification, the inert library was prepared for sequencing through PCR amplification of a 300 bp fragment (Inert_tagseq primer pair). The inert library was sequenced paired-end on an Illumina HiSeq X (~400 M reads) to acquire barcode and oligo pairings. dA-tailing, adaptor ligation, and indexing PCR amplification were completed by the sequencing centre (Custom Science).

#### Competent library

The inert library was then cloned into the pMPRAdonor2 vector using directional cloning (*Kpn*I and *Xba*I restriction enzymes), to form the final competent library backbone. The competent library was then transformed and purified, and QC steps were undertaken to confirm the correct sequence.

#### Primer sequences

MPRA_untailed_FWD_primer: 5’ – ACTGGCCGCTTGACG – 3’

MPRA_untailed_RVS_primer: 5’ – GCAGGAGCCGCAGTG – 3’

MPRA_SfiI_tailed_FWD_primer: 5’ – GCCAGAACATTTCTCTGGCCTAACTGGCCGCTTGACG – 3’

MPRA_SfiI_tailed_RVS_primer: 5’ – CCGACTAGCTTGGCCGCCGAGGCCCGACGCTCTTCCGATCT [N_**20**_ where N = A, T, C, G with equal chance of incorporation] TCTAGAGGTACCGCAGGAGCCGCAGTG – 3’

Inert_tagseq_FWD_primer: 5’ [N_4_ where N = A, T, C, G with equal chance of incorporation]GGCCT AACTGGCCGCTTGAC – 3’

Inert_tagseq_RVS_primer: 5’ – CCGCCGAGGCCCGACGCTCT – 3’

Barcode_seq_FWD_primer: 5’ – CAAGAAGGGCGGCAAGAT – 3’

Barcode_seq_RVS_primer: 5’ – CCGACGCTCTTCCGATCT – 3’

### Cell culture & transfection

HEK293 cells were cultured (CO_2_ = 5%; 37^o^C) in DMEM (Life Technologies #11965092) supplemented with 10% FBS. Cells were passaged every 2–3 days at ~80–90% confluency. Cell viability was measured using the Countess® II FL Automated Cell Counter and maintained at ~95% live cells. The competent library was transfected into HEK293 cells (in triplicate) using Lipofectamine-3000, in 2× T175 flasks, with cells at ~50–60% confluency. The transfection efficiency was determined by a separate mCherry transfection and visualisation.

### RNA (cDNA) & DNA processing

At 24 h post-transfection, cells were trypsinized and pelleted and DNA and RNA were harvested using the Qiagen All-prep DNA/RNA extraction kit (Qiagen; #80204), according to the manufacturer’s instructions. Following purification, the RNA was treated with DNase I (Qiagen; #79254) to remove any contaminating DNA. RNA integrity was assessed by visualisation following separation on a 1.2% agarose TBE gel. cDNA was then synthesised from the purified RNA using SuperScript III RT enzyme (Invitrogen; #18080400) and a custom- barcode specific primer (BSP). DNA and cDNA were then amplified for sequencing using NEBNext high-fidelity 2x master mix (NEB; #M0541S; Barcode_seq primer pair). DNA and cDNA were sequenced paired-end on an Illumina HiSeq X (Custom Science).

### RNA & DNA sequencing data processing, alignment & analysis (MPRA)

We developed a customised pipeline (Supplementary Fig. 4) to process the raw oligonucleotide sequencing data and to find barcodes within oligonucleotide, DNA and RNA sequencing libraries. Briefly, the pipeline trims adapter sequence (GGCCTAACTGGCCGCTTGACG) from the 5’ end of the oligonucleotide sequencing reads. The adapter trimmed reads were then aligned using *bwa* to a reference library consisted of the designed elements (Supplementary Table 1), without allowing indels and mismatches. In each perfectly aligned read, the 20 bp barcodes were identified using guide sequences “CGCCGAGGCCCGACGCTCTTCCGATCT” and “TCTAGAGGTACCGCAGGAGCCGCAGTG” flanking either side of the barcode. Alternatively, the 20 bp barcodes in the RNA and DNA sequencing libraries were detected by directly searching through the sequencing reads. In the RNA sequencing data, the barcodes were identified using the following guide sequences: i) TCTAGAATTATTACACGG attached at the end of the barcode in the forward reads and ii) “GTAATAATTCTAGA” and “AGATCGGAAGAGCGTC” flanking either side of the barcode in the reverse reads. Similarly, in the DNA sequencing data, the 20 bp barcodes were detected using the using the guide sequence i) “CCGACGCTCTTCCGATCT” and “TCTAGAATTATTACACGG” or “TCGCCGTGTAATAATTCTAGA” and “AGATCGGAAGAGCG”; and ii) “CCGACGCTCTTCCGATCT” and “TCTAGAATTATTACACGG” or “TCGCCGTGTAATAATTCTAGA” and “AGATCGGAAGAGCG” flanking either side of the barcode in the forward and reverse reads, respectively. Following the identification of barcodes, we counted the number of barcodes per variant and found that on average each variant mapped to ~400 barcodes (Supplementary Fig. 1). The mapped DNA and RNA barcodes were then aggregated for each variant. During the aggregation process we required that the barcodes were present across all 3 replicates. Following this process, we omitted any element that was represented by less than 5 barcodes.

#### General enhancers

To identify general enhancer elements, we determined the DNA:RNA ratio for each element and calculated Z-scores. Any element that that had a Z-score of 3 ($\pm$3 SD from the mean) was deemed to be an active enhancer.

#### Allele-specific enhancers

We used the *mpralm* Bioconductor package^22^ to identify allele-specific enhancer elements. *mpralm* is a linear model framework that enables the detection of differential activity between different alleles. The following parameters were used to run the pipeline: *“mpralm <- mpralm(object* = *mpraset, design* = *design, aggregate* = *“none”, block* = *block_vector, normalize* = *TRUE, model_type* = *“corr_groups”, plot* = *TRUE)”*. We defined elements as active enhancers that had an adj.*p-*value < 0.05 (RNA count difference between ref and alt allele), and suggestive enhancers that had an adj.*p-*value between 0.05 and 0.1.

### Variant annotation

#### Depletion rank

Halldorsson et al. developed a depletion rank (DR) and assigned a rank for each 500 bp window of the genome, as a metric to characterise sequence conservation based on variation. We leveraged this to determine the depletion rank of the variants included within the MPRA library. DR assignment was computed for an overlapping set of 500 bp windows in the genome with a 50 bp step size, thus meaning each variant will be linked to ~10 different DR scores. After overlapping SNPs with their respective DR scores, we took the mean of these scores.

#### Transcription factor binding site disruption

To predict if MPRA variants alter transcription factor binding sites (TFBS), we used the FABIAN prediction tool^26^. FABIAN is a web-based application that uses TFFMs and PWMs to predict the degree to which DNA variants are likely to disrupt (or create) the binding sites of TFs. For our analysis we selected only the TFFM models for prediction given they tend to be a better representation of TFBS when compared with PWMs. FABIAN provides one score per TFBS per variant, from 1 to −1, with 0 indicative of no disruption. A higher score indicates an increased binding affinity, and a lower score indicates a weakened binding affinity. FABIAN does not as such indicate any confidence thresholds and thus, we chose an arbitrary cut-off of $\pm$0.8 to subselect those that we deemed to be ‘high-confidence’ predictions.

#### LOEUF score

LOEUF (loss-of-function observed/expected upper bound fraction)^46^ scores were obtained from gnomAD v2.1.1^46^ (https://gnomad.broadinstitute.org/) to determine the level of constraint on *PARK* genes.

#### HaploReg epigenomic annotations

To predict if MPRA variants overlap with epigenomic and regulatory annotations, we ran the list of general enhancer and allele-specific enhancer variants through HaploReg v4.2 (https://pubs.broadinstitute.org/mammals/haploreg/haploreg.php).

#### Cell-type specific functional annotations

To understand the likely regulatory impact of the MPRA identified allele-specific variants within the brain, we used a number of different publicly available datasets to overlap the variants with epigenetic annotations within brain cell-types and tissues. We used the following datasets: FOUNDIN-PD: ATACseq peaks (i.e., open chromatin) and methylation marks observed during iPSC differentiation of dopaminergic neurons (day 0, 25, and 65)^31^; Nott et al.: ATACseq/H3K27ac/H3K4me3 epigenetic annotations in four brain cell types (neurons; microglia; astrocytes; oligodendrocytes)^32^*;* Fullard et al.: ATACseq peaks within the Brain Open Chromatin Atlas (BOCA)^33^; Corces et al.: bulk ATACseq peaks across seven brain regions^34^; and GTEx catalogue: eQTL annotations across 13 brain tissues^35^. Overlapping regions were identified by comparing the coordinates of the reported epigenetic annotation with the MPRA element coordinates (i.e., SNP coordinate +/−100 bp) using the ‘bedtools’ package. eQTLs within brain tissues were identified through the GTEx catalogue web interface.

### Precision gene editing of MPRA identified enhancer variants

#### Cell culture and CRISPR cas9 edits

The KOLF2.1J induced pluripotent stem cell (iPSC) line^37^ was used to develop isogenic clones that differed only at the variant of interest. KOLF2.1J cells were cultured (CO_2_ = 5%; 37 °C) in Stemflex media (Thermofisher #A3349401). Cells were passaged every 4–5 days at ~70–80% confluency using ReLeSR. Cell viability was measured using the Countess® II FL Automated Cell Counter and maintained at >90% live cells. To introduce the single base variants (rs11646653, rs78222414; rs3815082), we utilised the protocol previously described for precision gene editing using cas9 in the KOLF2.1J iPSC line^73,74^.

#### RNA extraction and sequencing

Following successful introduction of the desired edit and expansion of multiple clones, cells were lysed directly (in 24 well plates), and RNA was extracted using the RNeasy+ mini kit (Qiagen #74104), according to the manufacturer’s instructions. RNA integrity (i.e., RIN) was checked using the bioanalyzer, and all samples passed quality control. RNA was sequenced using the following parameters: stranded mRNA library prep & QC; 150 paired-end sequencing (Novaseq) to a depth of 30 M reads per sample.

#### RNA sequencing differential gene expression analysis

RNA-seq reads were quality checked using multiQC (v1.13)^75^ and mapped against the human reference genome GRCh38 using STAR (v 2.7.9a)^76^ aligner software. Following read alignment, we used featurecounts^77^ module from the Subread (v2.0.3) suite to count the number of reads mapping over individual genes. A matrix of gene counts per annotated gene was generated in Gencode project (v43) (https://www.gencodegenes.org/). The gene count matrix generated by the featurecounts program was then used as an input for an R package DESeQ2 (v1.42)^78^ and performed differential gene expression analysis using default settings. We defined differential expressed genes as those with absolute Log2 Fold change difference >1 and adjusted *p*-value < 0.01.

### Identification of PARK gene eQTLs

For each of the 21 *PARK* genes (Supplementary Table 3), we tested variants within their coding region for regulatory potential on modifying distant genes. We selected all common SNPs within the GENCODE gene coding region (including intronic regions; dbSNP build151, appear in at least 1% of the global population).

We also tested whether variants across the genome had a significant effect on the transcription any of the 21 *PARK* genes. We performed a genome-wide search of all 42,953,834 SNPs in dbSNP151 (as available in GTEx v8^79^) for an association with transcription of at least one of the 21 PD genes (Supplementary Table 3). All SNPs suggestive of genome-wide significance (*p* < 1 × 10^−6^) were also subsequently tested with the CoDeS3D algorithm^39^ to discover genes co-regulated by these SNPs.

For all variants tested in both analyses (gene locus and genome-wide), putative spatial regulatory connections were identified via the CoDeS3D algorithm^39^ (https://github.com/Genome3d/codes3d-v1). CoDeS3D integrates data on spatial interactions between genomic loci (Hi-C data) with expression data (genotype-tissue expression database version 8; GTEx v8) to identify genes whose transcript levels are associated with a physical connection to the SNP (i.e. spatial eQTL; Supplementary Table 4)^39,80^. The CoDeS3D method, and tissues and cell-types included, has been described in depth in previously^39,40^.

### Target gene assignment

The CoDeS3D^39^ algorithm wsas also used to identify genes whose transcript levels are putatively regulated by the MPRA-identified enhancer elements. Spatial-eQTLs were identified across all cell- and tissue- types.

### Reporting summary

Further information on research design is available in the Nature Research Reporting Summary linked to this article.

### Supplementary information

Reporting Summary

Supplementary Figures 1-7

Supplementary Tables 1-13

## Data Availability

MPRA summary data can be found in the supplementary tables at 10.17608/k6.auckland.24882012.v2. Raw output data is available upon request.

## Code availability

The MPRA and RNAseq data analysis pipelines are available at: https://github.com/sfar956/Parkinsons-disease-MPRA.git.
