# Supplementary material for: Identification of 27 allele-specific regulatory variants in Parkinson’s disease using a massively parallel reporter assay
Source: NPJ Parkinsons Dis. 2024 Feb 27;10:44. doi: 10.1038/s41531-024-00659-5 (PMC10899198; doi:10.1038/s41531-024-00659-5)
Supplement: Supplementary file 2 — Supplementary Figures 1-7 [file 41531_2024_659_MOESM2_ESM.pdf]

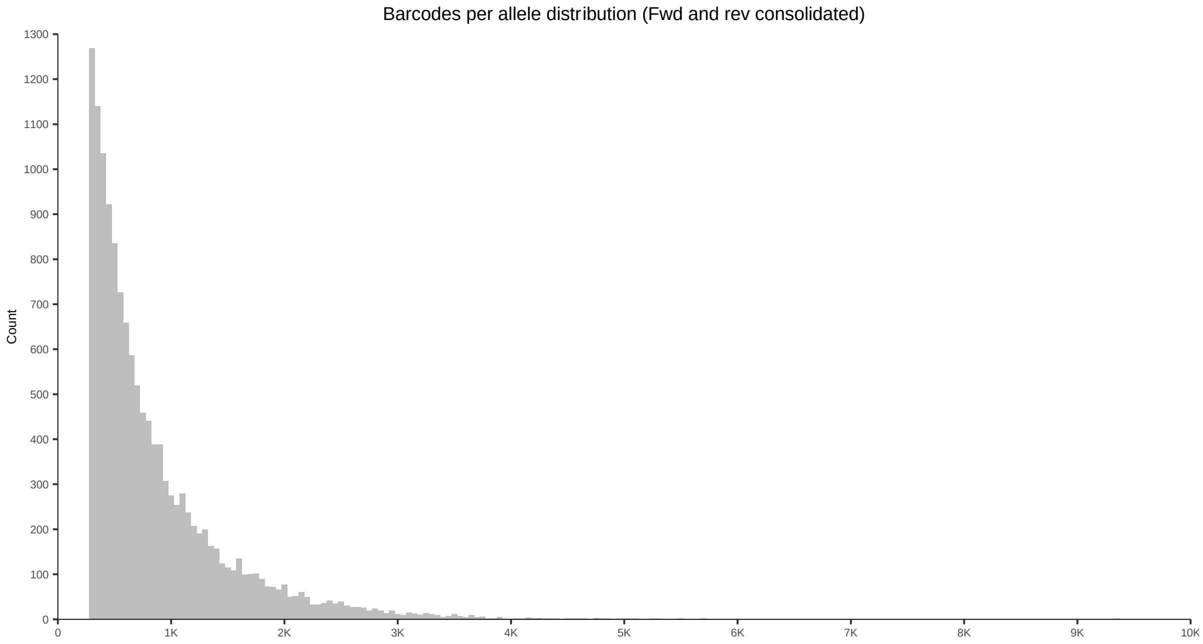

Supplementary figure 1: Number of tagging barcodes per element included in the library.

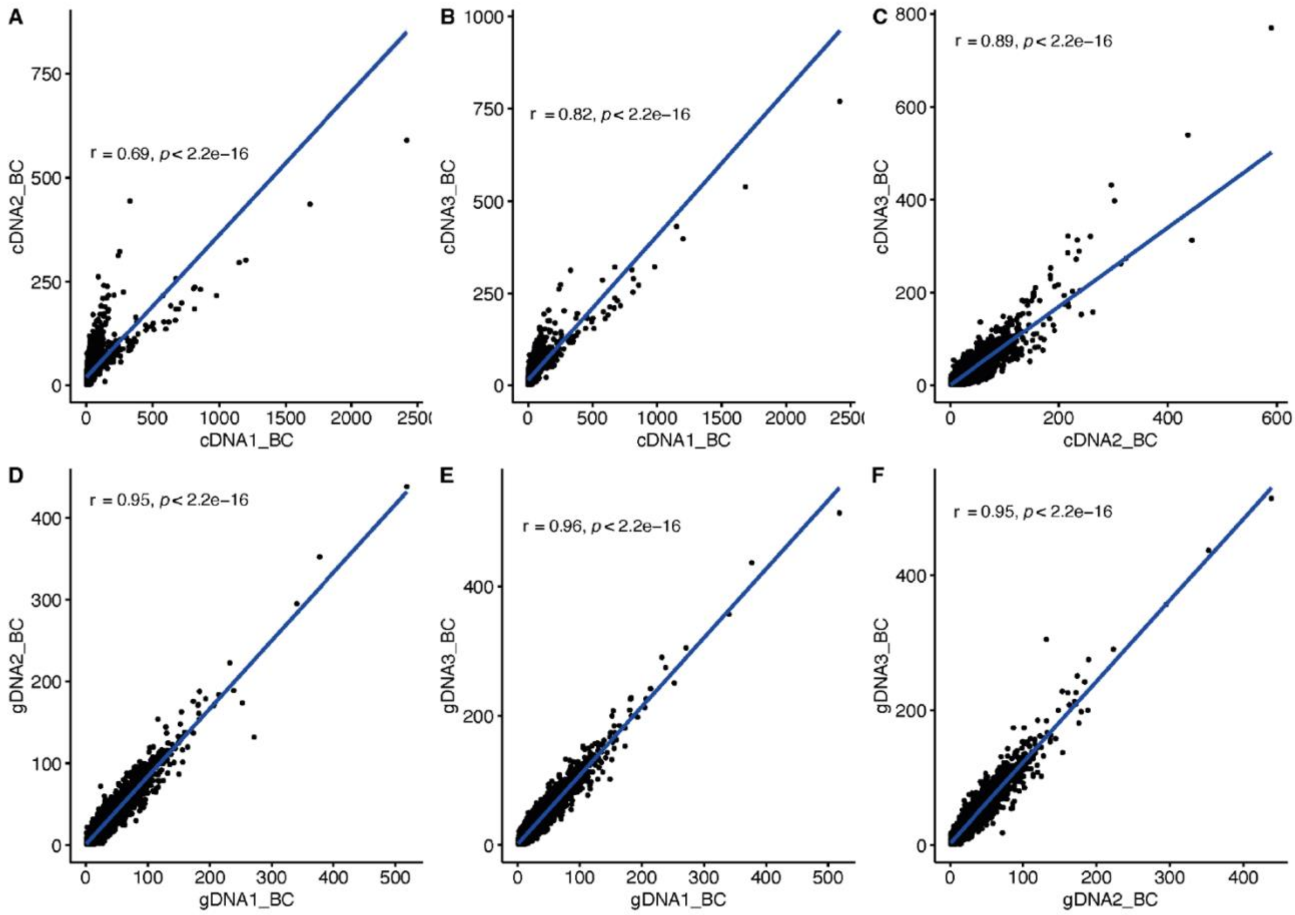

Supplementary figure 2: Correlation observed between transfection replicates in HEK293 cells for gDNA and cDNA.

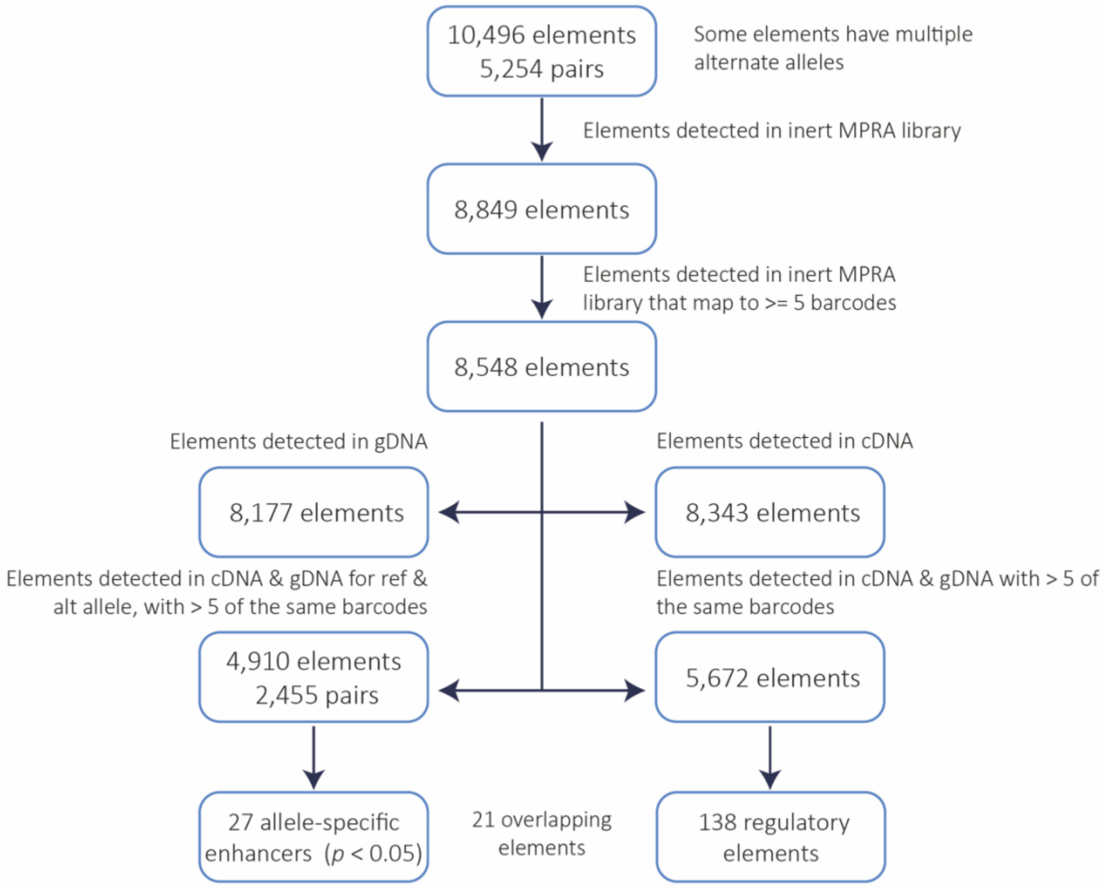

Supplementary figure 3: Number of elements included in the analysis pipeline at each stage.

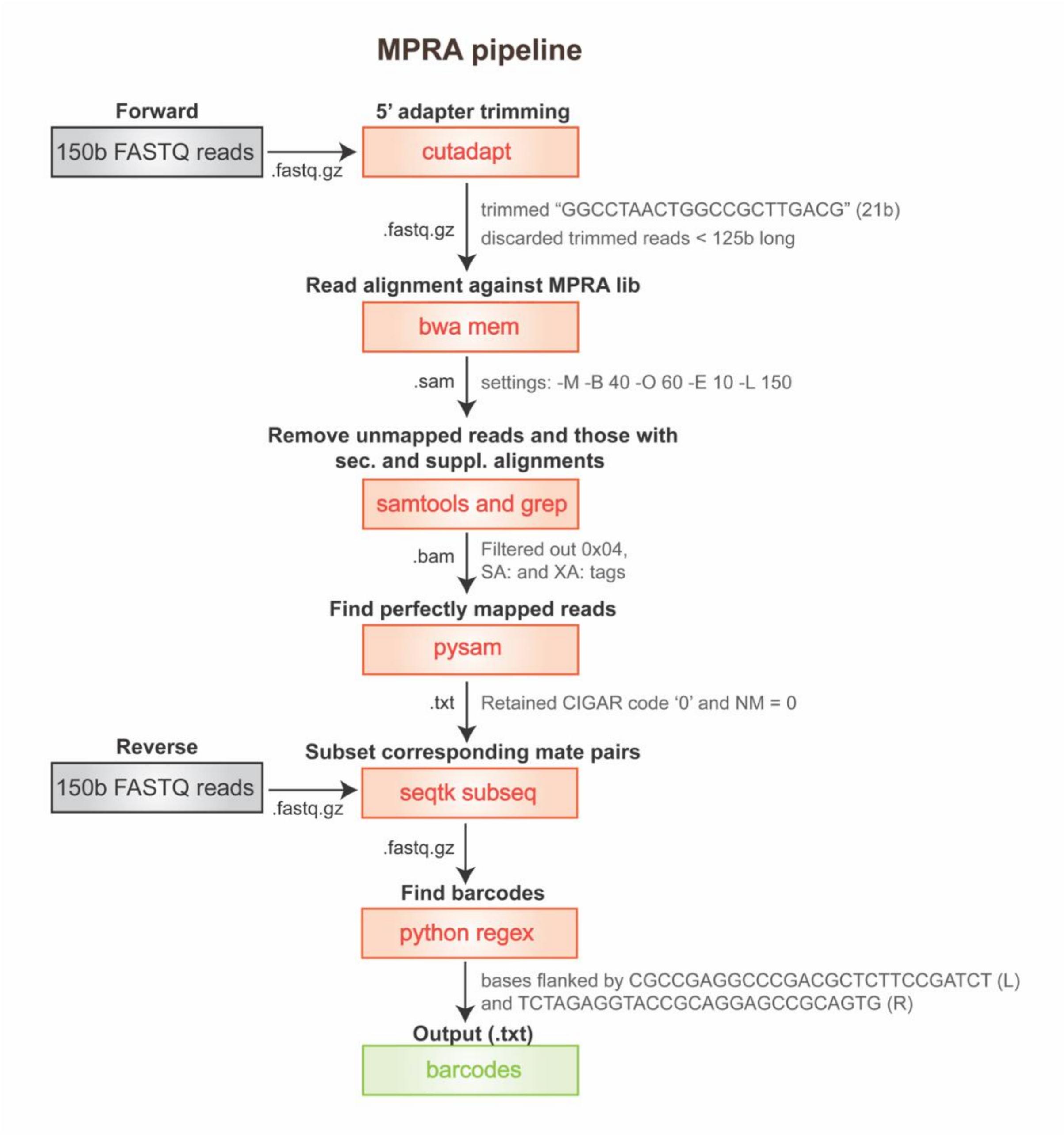

Supplementary figure 4: Sequencing analysis pipeline for aligning sequences within the library and mapping to their paired barcodes.

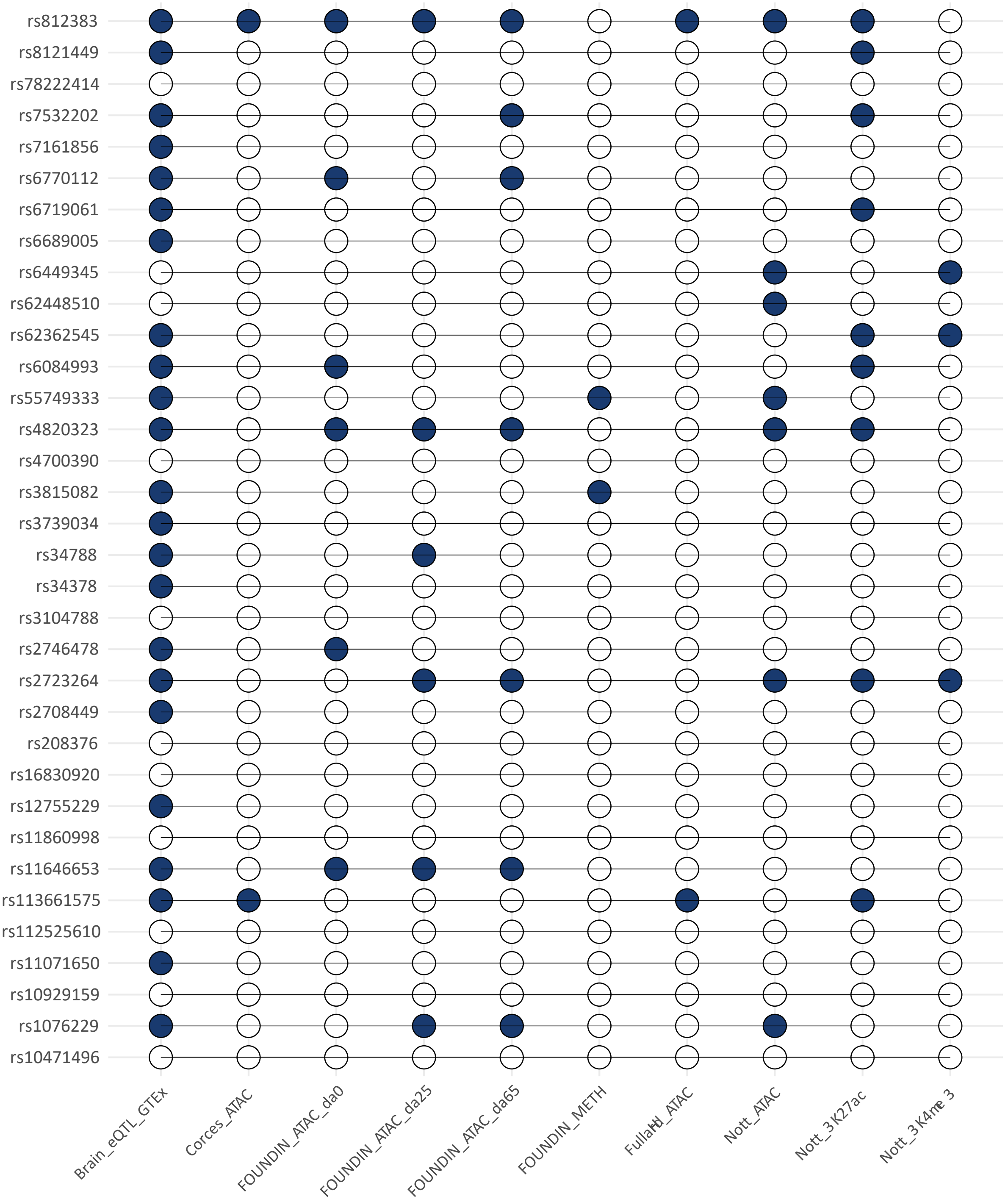

Supplementary figure 5: Bubble plot showing overlap between MPRA-identified allele specific enhancers and epigenetic annotations from publicly available datasets. Dark blue indicates overlap; white shows no overlap. Brain_eQTL_GTEx: SNP acts as an eQTL in at least 1 of the 13 brain tissues included in the GTEx database; FOUNDIN_ATAC_da[0,25,65]: ATACseq peaks (*i.e.,* open chromatin) observed during iPSC differentiation of dopaminergic neurons (day 0, 25, and 65), within the FOUNDIN-PD dataset; FOUNDIN_METH: Methylation marks observed in iPSC-derived dopaminergic neurons, within the FOUNDIN-PD dataset; Corces_ATAC: reproducible peak identified by bulk ATACseq across 7 brain regions; Fullard_ATAC: regions of open chromatin as identified within the Brain Open Chromatin Atlas (BOCA). Nott_ATAC/H3K27ac/H3K4me3: stated epigenetic annotation identified in at least one brain cell type (neurons; microglia; astrocytes; oligodendrocytes). All overlapping regions were identified by comparing the coordinates of the reported epigenetic annotation with the MPRA element coordinates (*i.e.,* SNP coordinate +/- 100bp) using the 'bedtools' package.

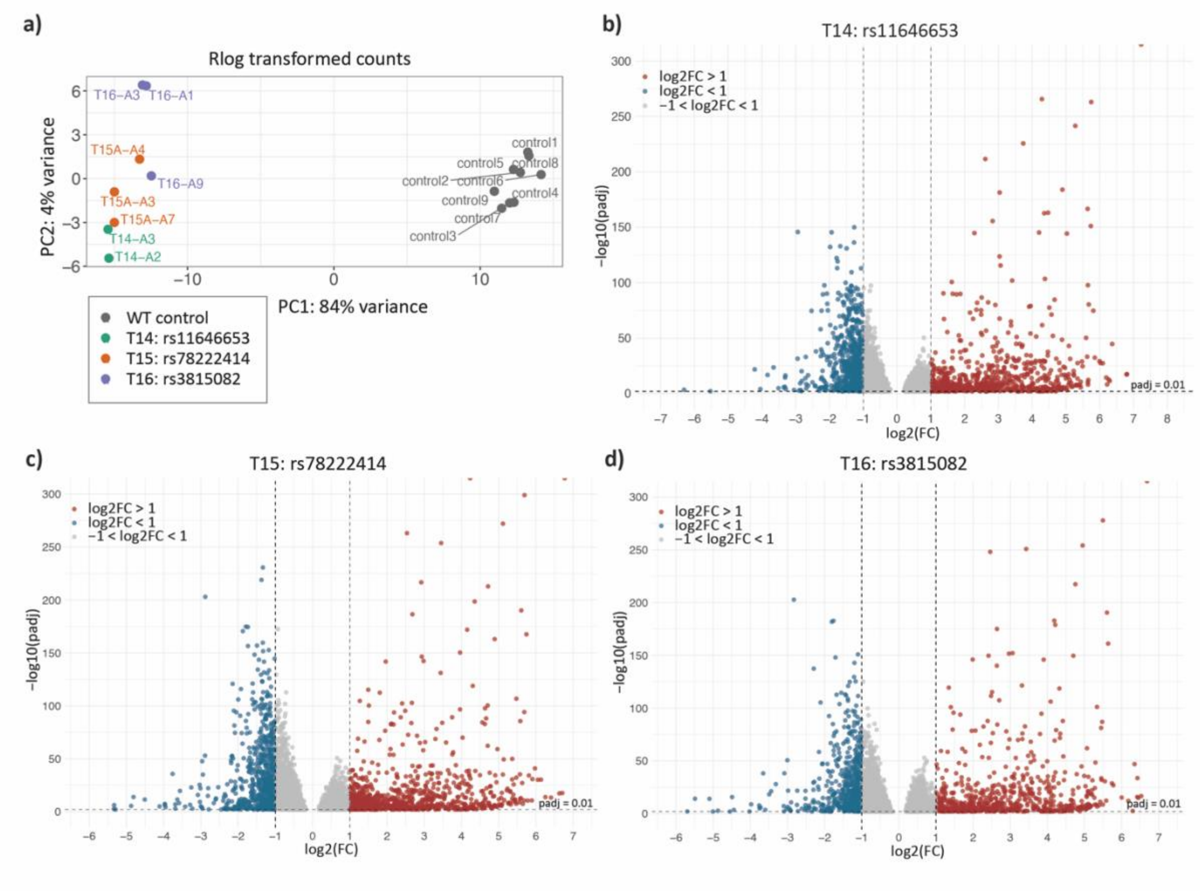

Supplementary figure 6: RNA sequencing differential gene expression analysis for three of the top allele-specific enhancer variants (rs11646653, rs78222414, rs3815082). a) PCA plot to show clustering of samples based on similarity. There is a large proportion of shared differentially expressed genes between the three targets, which likely arise due to the nucleofection process; b-d) volcano plots of differential gene expression in edited clones compared to WT control.

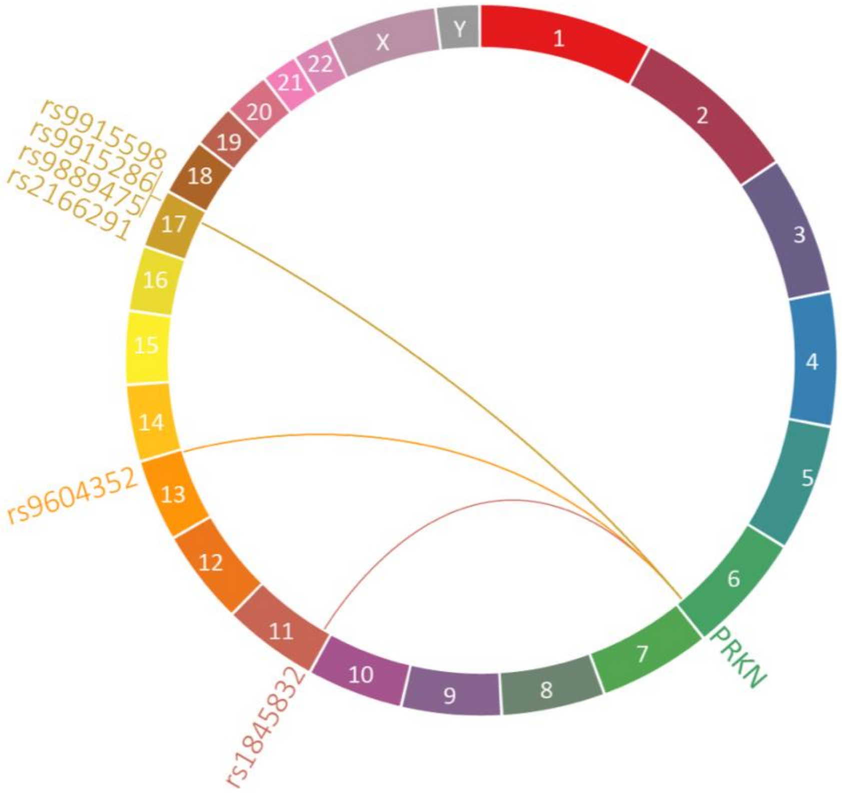

Supplementary figure 7: Circos interaction plot showing the MPRA-confirmed regulatory loci that have putatively been connected to the expression of *PRKN* (chr.6)
